# Rare variants in *SLC5A10* are associated with serum 1,5-anhydroglucitol (1,5-AG) in the Atherosclerosis Risk in Communities (ARIC) Study

**DOI:** 10.1038/s41598-019-42202-0

**Published:** 2019-04-11

**Authors:** Stephanie J. Loomis, Anna Köttgen, Man Li, Adrienne Tin, Josef Coresh, Eric Boerwinkle, Richard Gibbs, Donna Muzny, James Pankow, Elizabeth Selvin, Priya Duggal

**Affiliations:** 1 Department of Epidemiology, The Johns Hopkins University Bloomberg School of Public Health, Baltimore, MD USA; 2 Institute of Genetic Epidemiology, Medical Center and Faculty of Medicine - University of Freiburg, Freiburg, Germany; 3 Division of Nephrology and Department of Human Genetics, University of Utah, Salt Lake City, Utah USA; 4 Welch Center for Prevention, Epidemiology, & Clinical Research, The Johns Hopkins University, Baltimore, MD USA; 5 Department of Epidemiology, The University of Texas Health Science Center at Houston School of Public Health at Houston, Houston, TX USA; 6 Human Genome Sequencing Center, Baylor College of Medicine, Houston, TX USA; 7 Division of Epidemiology and Community Health, University of Minnesota, Minneapolis, MN USA

## Abstract

Serum 1,5-anhydroglucitol (1,5-AG) is an emerging biomarker used to monitor glycemic control in persons with diabetes. We performed whole-exome sequencing, examining the association between rare, coding genetic variants and 1,5-AG among European ancestry (N = 6,589) and African ancestry (N = 2,309) participants without diagnosed diabetes in the Atherosclerosis Risk in Communities (ARIC) Study. Five variants representing 3 independent signals on chromosome 17 in *SLC5A10*, a glucose transporter not previously known to transport 1,5-AG, were associated with 1,5-AG levels up to 10.38 µg/mL lower per allele (1,5-AG range 3.4–32.8 µg/mL) in the European ancestry sample and validated in the African ancestry sample. Together these variants explained 6% of the variance in 1,5-AG. Two of these variants (rs61741107, p = 8.85E-56; rs148178887, p = 1.13E-36) were rare, nonsynonymous, and predicted to be damaging or deleterious by multiple algorithms. Gene-based SKAT-O analysis supported these results (*SLC5A10* p = 5.13E-64 in European ancestry, validated in African ancestry, p = 0.006). Interestingly, these novel variants are not associated with other biomarkers of hyperglycemia or diabetes (p > 0.2). The large effect sizes and protein-altering, multiple independent signals suggest *SLC5A10* may code for an important transporter of 1,5-AG in the kidney, with a potential nonglucose-related effect on 1,5-AG, impacting its clinical utility as a diabetes biomarker in this subpopulation.

## Introduction

1,5-andhydroglucitol (1,5-AG) is an emerging biomarker of glycemic control in type 2 diabetes. 1,5-AG is a monosaccharide consumed in food and maintained at high, constant levels in the blood under normoglycemic conditions through filtration by the kidney and reabsorption into the blood. 1,5-AG is the 1-deoxy form of glucose, and during hyperglycemic conditions (i.e., when glucose exceeds the renal threshold), glucose outcompetes 1,5-AG for reabsorption. This causes 1,5-AG excretion in urine and hence lower levels in blood concentrations^1^. In adults with diabetes, low 1,5-AG concentrations reflect glucose excursions over the previous 2–14 days^1,2^, and are associated with microvascular and macrovascular disease^3–5^.

In a recent genome-wide association study (GWAS), we identified seven variants at six loci associated with 1,5-AG among persons of European ancestry without diagnosed diabetes^6^. Two of these variants were also found in a genetic screen of 1,5-AG measured as part of a large non-targeted metabolome panel among Europeans^7^. These variants map in or near genes which are involved in carbohydrate metabolism (*LCT, SI, MGAM, MGAM2*) and glucose transport in the gut and kidney (*SLC5A10, SLC50A1, SLC5A1*)^6^. Interestingly, the majority of the variants are not associated with traditional measures of hyperglycemia such as fasting glucose and HbA1c^8,9^.

While array-based analyses such as GWAS often capture common variants in linkage disequilibrium with the putative causal variants, they are not able to assess the impact of rare variants, nor are they designed to identify causal variants. To investigate the association of rare, putatively damaging variants with 1,5-AG, and to further understand the genetic architecture of this biomarker, a whole-exome sequencing association study of 1,5-AG concentrations was performed in the Atherosclerosis Risk In Communities (ARIC) Study.

## Materials and Methods

### Study population

The ARIC Study is an ongoing, longitudinal cohort study initiated in 1987, when middle-aged adults were recruited from four communities in the U.S.: Forsyth, North Carolina; suburban Minneapolis, Minnesota; Washington County, Maryland and Jackson, Mississippi. A total of 15,792 individuals attended the initial study visit (1987–1989), and subsequent visits occurred in 1990–1992 (visit 2), 1993–1995 (visit 3), 1996–1998 (visit 4), and 2011–2013 (visit 5), and 2016–2017 (visit 6) with a seventh visit ongoing. The study protocol was approved by the Institutional Review Boards (IRB) of all participating institutions: University of North Carolina at Chapel Hill IRB, Wake Forest University IRB, Johns Hopkins University IRB, University of Minnesota IRB, and University of Mississippi Medical Center IRB. The study was conducted in accordance with the Declaration of Helenski, and all study participants provided written informed consent^10^. For this study, data from individuals who attended visit 2 (N = 14,348) was used.

### 1,5-AG measurement

1,5-AG concentrations were measured using a colometric assay where 1,5-AG is oxidized to hydrogen peroxide (GlycoMark, Winston-Salem, NC) by the Roche Modular P800 system. Serum was collected at visit 2 (1990–1992) and analyzed in 2012–2013. The interassay coefficient of variation was 4.8%^11^ and the reliability coefficient was 0.99, calculated from 610 duplicate pairs of samples. Previous studies have shown high reliability in samples stored for long periods of time^12^.

### Exome sequencing

DNA was extracted from blood primarily collected at visit 1. All sequencing was performed as part of the CHARGE Consortium exome sequencing project at the Baylor College of Medicine Human Genome Sequencing Center (HGSC). Samples were bar-coded, pooled and sequenced using paired-end sequencing, run on the Illumina HiSeq 2000 or 2500 platform (San Diego, CA), and exome capture performed with VCRome 2.1 (NimbleGen, Inc., Madison, WI). Sequence alignment was done using the Burrows-Wheeler alignment^13^ tool with the Genome Reference Consortium Human Build 37 reference sequence. Aligned reads were then recalibrated using the Genome ANalysis ToolKit (GATK). Variant calling was done with the Mercury pipeline (https://www.hgsc.bcm.edu/content/mercury) in DNAnexus. VCF files were generated using the Atlas2 suite (Atlas-SNP and Atlas-Indel).

### Quality Control

Standard quality control exclusion measures were implemented to ensure accurate, reliable results. Single nucleotide variants (SNVs) were excluded if they met any of the following criteria: posterior probability <0.95, variant read count <3, variant read ratio <0.25 or >0.75, strand bias >99% in single direction, total coverage <10 fold for SNVs (<30x for indels), outside exon capture regions, monomorphic variant, missing rate >20%, mappability score <0.8, mean depth coverage >500 fold, Hardy Weinberg Equilibrium p < 5 × 10^−6^ in ancestry-specific groups. All samples provided consent for use in DNA studies and had adequate DNA for exome sequencing analysis. Samples were excluded if they had >20% missing data or fell less than 6 standard deviations (SD) from mean read depth, more than 6 SD for singleton count, outside of 6 SD for heterozygote to homozygote ratio or transition to transversion (Ti/Tv) ratio. After quality control, 2,556,859 SNVs and 76,133 indels remained, and 7,810 European Ancestry individuals and 3,180 African Ancestry individuals remained. Individuals who did not attend visit 2 (N = 594), were missing diabetes status (N = 2), had diagnosed diabetes (self-reported physician diagnosis or use of diabetes medications; N = 875), or missing 1,5-AG data at visit 2 (N = 621; Supplementary Fig. S1) were also excluded. In total, 6,589 European ancestry samples and 2,309 African ancestry samples were analyzed.

### Variant annotation and functional prediction

ANNOVAR8 and dbNSFP v2.0 (https://sites.google.com/site/jpopgen/dbNSFP) were used to annotate variants to genes and functional predictions using the GRCh37 reference sequence and National Center for Biotechnology Information RefSeq. Functional annotation by several metrics predicted if a variant was expected to be damaging (an amino acid change which negatively impacts protein function) or deleterious (a variant which reduces fitness and is subject to purifying selection). SIFT score predicts if an amino acid change is likely to be damaging to protein function based on conservation (i.e., well conserved regions are assumed to be biologically important and thus variants in these regions are more likely to be damaging)^14^. A SIFT score <0.05 was considered damaging. Polyphen-2 flags amino acid changes that are predicted to be damaging based on the structure and function of a protein. Polyphen-2 score >0.957 was considered damaging, and a score between 0.453 and 0.965 was considered possibly damaging^15^. GERP predicts substitutions that would have occurred if the region was not under selection and quantifies rate of substitutions that did not occur^16^. A GERP score >2 was considered deleterious. Finally CADD aggregates annotations of allelic diversity, functionality, pathogenicity, disease severity, regulatory effects, complex trait associations, and known pathogenic variants into a score^17^. A CADD score >15 was considered deleterious. In addition, the Bravo portal (https://bravo.sph.umich.edu/freeze5/hg38/) was used to obtain TOPMed and 1000 Genomes allele frequencies (Freeze 5, including 463 million variants on 62,784 individuals). For each significant variant, the Genotype-Tissue Expression (GTEx) Project was searched for expression quantitative trait loci (eQTLs; https://www.gtexportal.org/home/).

### Single-variant tests

Genetic associations with 1,5-AG were analyzed using both single-variant and gene-based tests using the R package SeqMeta. All analyses were run separately by ancestry. 1,5-AG values were winsorized at 1% and 99% to account for long tailed distributions. Single variant analyses were run as linear regressions controlling for age, sex, ARIC study center and significantly associated principal components (p < 0.05; N = 2 for European ancestry, 1 for African ancestry). To ensure our results were not driven by a very small number of individuals, variants with less than 10 copies of the minor allele (minimum minor allele frequency (MAF) = 10/(N*2)) were excluded. For the European ancestry sample MAF <0.008 (10/(6,589*2)) was used and for the African ancestry sample MAF <0.002 (10/(2,309*2)) was used. A Bonferroni correction to calculate a statistical significance threshold as 1.4 × 10^−7^ (0.05/121,052 variants) was used for European ancestry and 2.8 × 10^−7^ (0.05/175,583 variants) was used for African ancestry. Variance explained by individual variants was calculated as the difference between the coefficient of determination from the null model (the association between 1,5-AG and covariates) and a model adjusting for effect of the variant controlling for the same covariates.

### Gene-based tests

To augment power for situations where multiple rare variants affect association with a phenotype, the SKAT-O test was run, which aggregates variants into genes and tests for association between genes and phenotypes. SKAT-O combines a burden test, which has greater power when variants are associated with the phenotype and in the same direction, with SKAT, a kernel based, variance components test, which has greater power when fewer variants are causal or affect risk in both directions. Genes with ≤1 variant per gene were excluded. Variants were not filtered by MAF in the main analysis. Variants other than nonsynonymous, splicing, stop-gain, stop-loss, or frameshift were excluded from the association analyses. Additionally, genes in which all variants used for the burden test together had a cumulative MAF <0.005 were excluded. A Bonferroni correction was used to calculate a significance threshold: 4.0 × 10^−6^ (0.05/12,504 genes) for European ancestry and 3.3 × 10^−5^ (0.05/14,499 genes) for African ancestry. Secondary analyses of SKAT and a T1 burden test (where all variants with MAF <0.01 were collapsed into a score for each gene) were also done.

### Conditional analyses

To determine if the single-variant results represent independent signals, the top variant (defined as most significant and most deleterious or damaging by GERP, SIFT, Polyphen2 and CADD) was conditioned on for each locus with multiple significant variants. Secondary conditioning analyses were also performed on the most significant variant and the previous GWAS-identified variant. Regional association plots using LocusZoom (http://locuszoom.org/)^18^ were created to visualize the region prior to and after conditioning on the top variants.

### Variant association with diabetes

To determine if variants significantly associated with 1,5-AG also impact diabetes, the association between these variants and prevalent diabetes status was evaluated. This analysis was performed for both diagnosed diabetes (self-reported physician diagnosis or use of diabetes medications) and the combination of diagnosed diabetes and undiagnosed diabetes (defined as fasting glucose ≥126 mg/dL if fasting for ≥8 hours or non-fasting glucose ≥200 mg/dL).

## Results

### Study population

There were 6,589 individuals in the European ancestry sample and 2,309 individuals in the African ancestry sample. In both groups, over half were female, and mean age was 56 to 57 years old. 1,5-AG levels were lower, and fructosamine, glycated albumin, fasting glucose and HbA1c were higher in the African ancestry sample as compared to the European ancestry sample. Study population characteristics are detailed in Supplementary Table S1.

### Single variant and gene-based analyses

In the European ancestry sample, 15 variants reached exome-wide significance for association with 1,5-AG in single variant testing (Table 1). These variants are located in 6 loci on chromosomes 1, 2, 3, 7, 17, and 22, all of which were also identified in our previous GWAS of 1,5-AG concentrations^6^. None of the African ancestry single variant or gene-based results were statistically significant.

**Table 1:** Significant^a^ 1,5-AG (µg/mL) single SNP results in European ancestry sample, with validation in the African ancestry sample.

SNP	Gene	Chr	A1/A2^b^	Function^c^	Amino acid change	GERP, SIFT, Poly-phen2, CADD Prediction^d^	TOP Med AF	TGP EA Effect AF^e^	TGP AA Effect AF^e^	European ancestry (N = 6,589)				African ancestry (N = 2,309)			
										Effect AF	Beta (SE)	P-value	% Var. Explained	Effect AF	Beta (SE)	P-value	% Var. Explained
rs61741107	*SLC5A10*	17	G/A	**NS**	G > E	**D,D,D,D**	0.004	0.002	0	0.007	−9.31 (0.59)	8.85E-56	2.95	0.0005^g^	−9.17 (3.95)	0.02	0.26
rs148178887	*SLC5A10*	17	A/T	**NS**	N > I	**D,D,D,D**	0.002	0.005	0	0.004	−10.38 (0.82)	1.13E-36	1.71	0.002^g^	−9.93 (2.80)	3.83E-04	0.36
rs201046878	*SLC5A10/ FAM83G* ^f^	17	G/A	**NS**/I^f^	R > W	**D,D,D,D**	0.002	0.005	0.002	0.004	−8.33 (0.74)	1.96E-29	1.25	0.002^g^	−9.93 (2.80)	3.83E-04	0.33
rs200038747	*SLC5A10/ FAM83G* ^f^	17	C/T	**NS**/I^f^	R > Q	**D,D,D,D**	0.002	0.001	0.005	0.002	−9.04 (1.23)	1.69E-13	0.61	0.004	0.25 (1.25)	0.84	0.09
rs117355297	*SLC5A10*	17	C/T	S	—	T,NA,NA,**D**	0.022	0.05	0.001	0.04	−2.73 (0.26)	3.85E-26	1.37	0.005	−3.34 (1.12)	2.91E-03	0.2
rs4072037	*MUC1*	1	C/T	**NS**	—	T,NA,NA,T	0.60	0.55	0.60	0.54	−0.49 (0.10)	3.74E-07	0.26	0.67	−0.22 (0.18)	0.21	0.1
rs961360	*R3HDM1*	2	A/G	**NS**	M > V	**D**,T,B/**P**,T	0.22	0.23	0.25	0.15	−0.80 (0.14)	7.82E-09	0.32	0.20	−0.41 (0.21)	0.05	0.3
rs10445686	*RAB3GAP1*	2	A/G	**NS**	N > S	**D**,T,B,T	0.14	0.19	0.02	0.13	−0.79 (0.14)	3.59E-08	0.35	0.03	0.21 (0.49)	0.66	0.04
rs2304371	*LCT*	2	G/A	S	—	**D**,NA,NA,T	0.70	0.75	0.41	0.83	0.89 (0.13)	6.74E-12	0.49	0.45	0.39 (0.16)	0.02	0.13
rs3739022	*LCT*	2	G/A	S	—	T,NA, NAT	0.16	0.15	0.22	0.10	−1.07 (0.17)	1.23E-10	0.51	0.21	−0.48 (0.20)	0.02	0.09
rs1050115	*UBXN4*	2	A/G	S	—	T,NA,NA,T	0.17	0.21	0.17	0.15	−0.80 (0.14)	5.69E-09	0.35	0.14	−0.61 (0.24)	0.01	0.19
rs9283633	*SI*	3	T/C	**NS**	T > A	T,T,B,T	0.58	0.63	0.46	0.61	0.52 (0.10)	2.03E-07	0.33	0.48	0.29 (0.17)	0.09	0.17
rs185053832	*MGAM*	7	C/A	**NS**	P > T	**D,D,D,D**	0.006	0.01	0.001	0.01	−3.30 (0.49)	1.70E-11	0.63	0.002^7^	−1.26 (3.22)	0.69	0.01
rs17683011	*SLC5A1*	22	A/G	**NS**	N > S	T,T,B,T	0.04	0.06	0.003	0.07	−0.96 (0.19)	3.36E-07	0.31	0.02	−0.94 (0.66)	0.15	0.01
rs17683448	*SLC5A1*	22	C/T	S	—	T,NA,NA,T	0.04	0.06	0.003	0.06	−1.14 (0.21)	5.26E-08	0.38	0.01	−0.97 (0.71)	0.17	0.02

^a^Bonferroni corrected significance threshold = 4.1 × 10^−7^ (0.05/121,052 SNPs).

^b^A2 is effect allele.

^c^NS = nonsynonymous, S = synonymous, I = intron.

^d^GERP and CADD prediction: D = deleterious, T = tolerated otherwise; Polyphen2 and SIFT prediction: D = probably damaging, P = possibly damaging, B = benign.

^e^TGP = 1000 genomes allele frequency for Eur (EA) and Afr (AA), AF = allele frequency.

^f^*SLC5A10* and *FAM83G* are overlapping genes. These variants are missense variants in *FAM83G* and intronic to *SLC5A10*.

^g^Variants have minor allele count <1.

### Rare, deleterious variants on chromosome 17

Four rare (MAF <0.007) and one low frequency (MAF = 0.04) variants in the region of two overlapping genes on chromosome 17, *SLC5A10* and *FAM83G* (Table 1) were associated with 1,5-AG. Of the four rare variants, two were nonsynonymous to *SLC5A10* and were highly significant (rs148178887, p = 1.13 × 10^−36^ and rs61741107, p = 8.85 × 10^−56^). In addition, the effect sizes of these variants were large, approximately 10 µg/mL per risk allele, and explained 1.71% and 2.95% of the variance in 1,5-AG concentrations, respectively. The other two rare variants were intronic to *SLC5A10* but nonsynonymous to *FAM83G* (rs200038747, p = 1.69 × 10^−13^ and rs201046878, p = 1.96 × 10^−29^). The low frequency variant (rs117355297, p = 3.85 × 10^−26^) was also found in the GWAS^6^ and was synonymous to *SLC5A10*. All four nonsynonymous variants were predicted to be damaging or deleterious by the prediction programs GERP, Polyphen-2, SIFT and CADD. In addition, the nonsynonymous variants resulted in amino acid changes which altered polarity and acidity (for example, rs61741107 resulted in a change from nonpolar glycine to acidic glutamic acid, and rs148178887 resulted in a change from polar asparagine to nonpolar isoleucine). Four variants were also nominally (p < 0.05) associated with 1,5-AG in African ancestry individuals. The gene-based SKAT-O test showed significance for *SLC5A10* and *FAM83G* (Table 2). Secondary analyses of separate SKAT (p = 2.8 × 10^−55^), T1 burden (p = 2.5 × 10^−114^) and SKAT-O restricting variants to MAF <0.05 (p = 5.1 × 10^−64^) tests also showed strong significance for *SLC5A10* (Supplementary Tables S2–4).

**Table 2:** Significant^a^ 1,5-AG (µg/mL) gene-based results in European ancestry sample, validated in African ancestry sample.

Chr	Gene	European ancestry (N = 6,589)			African ancestry (N = 2,309)		
		P-value	cMAF^b^	N SNPs	P-value	cMAF	N SNPs
17	*SLC5A10*	5.13E-64	0.04	58	0.006	0.29	28
17	*FAM83G*	6.24E-17	0.06	56	0.39	0.34	36
7	*MGAM*	8.20E-07	0.09	148	0.06	0.95	98
22	*SLC5A1*	1.10E-06	0.23	48	0.21	0.07	15

^a^Bonferroni corrected significance threshold = 4.0 × 10^−6^ (0.05/12,504 genes).

^b^cMAF = cumulative minor allele frequency.

**Figure 1:**
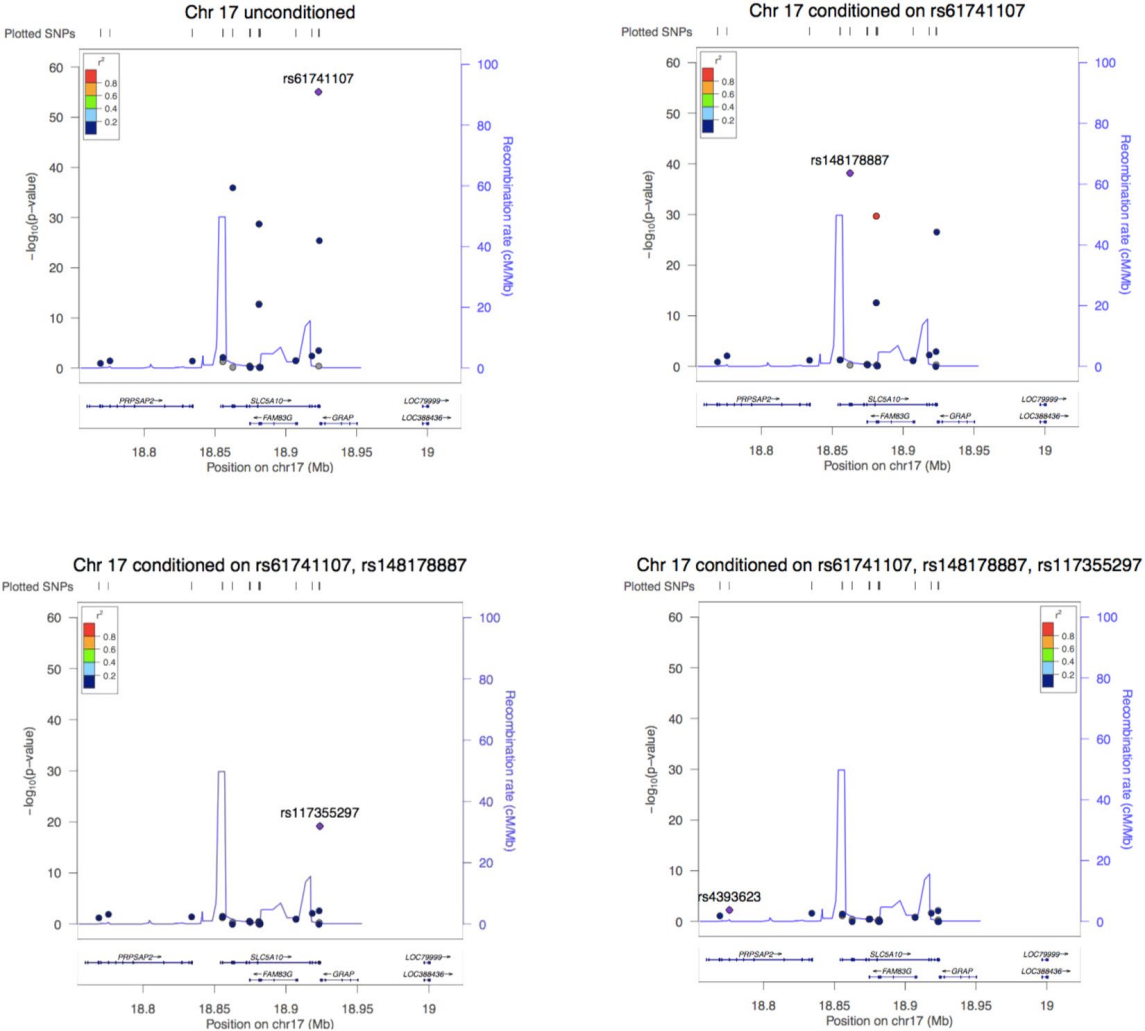
Regional association plots for top hits on chromosome 17 in European ancestry sample, unconditioned and conditioned. Unconditioned analysis shows five genome-wide significant hits. Conditioning on rs61741107 results in four remaining genome-wide significant hits. Conditioning on both rs61741107 and rs148178887 results in a single genome-wide significant hit, indicating that three of the previous variants represent a single signal. Conditioning on rs61741107 and rs148178887 and rs117355297 results in no genome-wide significant hits, providing evidence that the five significant variants in this region are represented by three independent signals.

To determine if the variants in this region were representing one signal in linkage disequilibrium or several independent signals, the nonsynonymous variants were conditioned on (Fig. 1). After conditioning on rs61741107, the variants rs148178887, rs201046878, rs200038747 and rs117355297 remained significant. After additionally conditioning on rs148178887, only rs117355297 remained significant. Further conditioning on the synonymous variant, rs117355297, produced no significant variants in this region. Secondary conditioning on the previously identified GWAS variant (rs117355297) showed the rare variants remained significant (p < 2.7 × 10^−14^). This suggests that these variants represent three independent, significant loci, which together explain 6% of the variance in 1,5-AG (Table 1). GTEx did not show eQTLs for any of the chromosome 17 variants in diabetes-relevant tissue such as the kidney, liver or pancreas.

**Figure 2:**
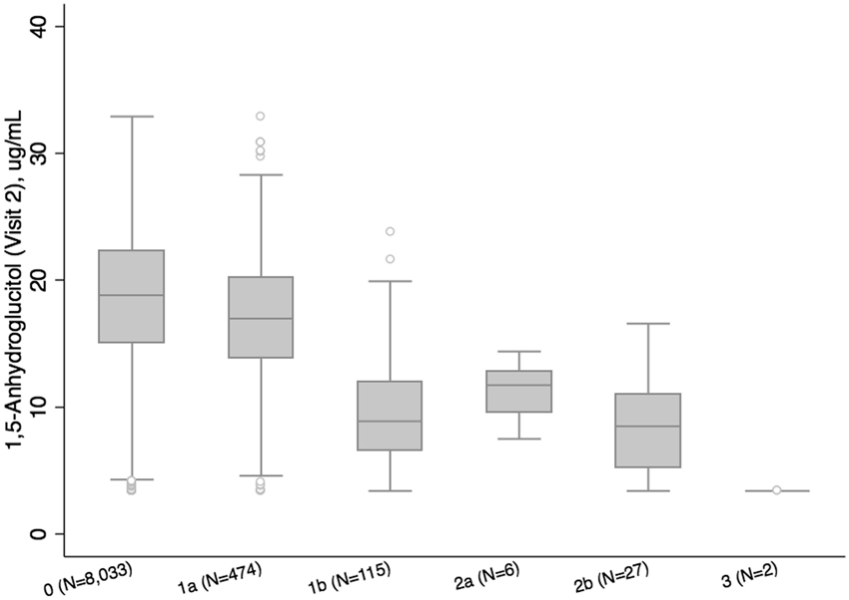
Distribution of 1,5-AG by chromosome 17 variants. a,b With each additional copy of a significant chromosome 17 risk allele, median 1,5-AG values decrease indicating a dose-response effect. a 1,5-AG is winsorised at 1% and 99%. Legend: 0: no copies of rs61741107, rs148178887 or rs117355297 minor alleles. 1a: 1 copy of rs117355297 minor allele. 1b: 1 copy of rs61741107 or rs148178887 minor alleles. 2a: 2 copies of rs117355297 minor alleles. 2b: 1 copy of rs61741107 or rs148178887 + 1 copy of rs117355297 minor allele. 3: 1 copy of rs61741107 or rs148178887 + 2 copies of rs117355297 minor allele.

To further explore the rare variants in chromosome 17, they were evaluated for an association with diabetes. Of the variants representing the three significant signals on chromosome 17, none were significantly associated with diagnosed or diagnosed + undiagnosed diabetes status in either European or African ancestry samples (Supplementary Table S5). In addition, mean 1,5-AG levels differed substantially between individuals with and without the chromosome 17 variants, while the mean values of other glycemic biomarkers did not. (Fig. 2, Supplementary Fig. S2). No individuals were homozygous for rs61741107 or rs148178887, but eight people were homozygous for rs117355297 (Supplementary Fig. S3). Four individuals had both rs61741107 and rs117355297 (mean 1,5-AG = 2.8 µg/mL, SD = 1.3 µg/mL), 23 had both rs148178887 and rs117355297 (mean 1,5-AG = 9.5 µg/mL, SD = 3.8 µg/mL), and two people were heterozygous for rs148178887 and homozygous for rs117355297 (mean 1,5-AG = 2.8 µg/mL, SD = 0 µg/mL; Fig. 2).

### Additional regions of interest

One common variant in *MUC1* on chromosome 1 in linkage disequilibrium (LD) with a variant identified in our GWAS (rs9330264, r^2^ = 0.5) was associated with 1,5-AG, but the gene-based test was not significant. Neither the single variant or gene-based test was validated in the African ancestry sample. GTEx indicated possible eQTLs in diabetes-related tissues (liver: *GBAP1* p = 1.6 × 10^−11^, *THBS3* p = 2.5 × 10^−6^; pancreas: *GBAP1* p = 3.3 × 10^−25^, *THBS3* p = 5.2 × 10^−11^, *GBA* p = 6.3 × 10^−8^).

Five common variants in genes *LCT, RAB3GAP1, R3HDM1*, and *UBXN4* were associated on chromosome 2 across a large region spanning 0.7 Mb. Three of these variants (rs961360, rs1050115, rs2304371) were in LD with the GWAS index variant, rs182549 (r^2^ = 0.27 to 0.35 in 1000 genomes phase3v5 European population). Two of the five variants were nonsynonymous, one of which (rs961360) was predicted to be possibly damaging by Polyphen-2. The remaining three variants were synonymous and one was also associated in African ancestry individuals (rs1050115, p = 0.01). Conditional analysis on the top nonsynonymous variants revealed two distinct signals in this region (Supplementary Table S6). The GWAS index variant was not present in this dataset and hence could not be conditioned on. None of the genes in this region were associated with 1,5-AG in the gene-based test.

One common variant on chromosome 3 in *SI* was associated with 1,5-AG in European ancestry individuals. This variant is in near perfect LD with the GWAS index variant, rs9825346 (r^2^ = 0.98). It is a nonsynonymous variant, but was not predicted to be damaging or deleterious by any of the prediction programs and was not significant in African ancestry individuals.

One low-frequency (MAF = 0.01) variant on chromosome 7 in *MGAM* was associated with 1,5-AG. This variant is not in LD with the GWAS index variant. It is nonsynonymous and predicted to be damaging or deleterious by GERP, Polyphen-2, SIFT and CADD, but was not significant in African ancestry individuals. *MGAM* was associated with 1,5-AG in the gene-based test.

Finally, two common variants in *SLC5A1* were associated in this region. Both variants were in near perfect LD with each other and the GWAS index variant, rs117086479 (r^2^ = 0.98 to 1). One variant was nonsynonymous (rs17683011) and the other was synonymous (rs17683448). Neither had evidence for deleteriousness by any measure, and neither variant was associated with 1,5-AG in African ancestry individuals. *SLC5A1* was significant in the gene-based test.

## Discussion

In this exome sequencing analysis, 15 variants were significantly associated with 1,5-AG among people of European ancestry without diabetes, and four of these variants in two loci were validated in a sample of African ancestry individuals. In addition, 4 genes were associated with 1,5-AG among individuals of European ancestry, of which one (*SLC5A10*) validated in the African ancestry sample.

Both single variant and gene-based tests identified a region on chromosome 17 in or near *SLC5A10* and the overlapping gene, *FAM83G. SLC5A10* is a glucose transporter exclusively expressed in the kidney^19^, and is not known to also transport 1,5-AG. Our results, however, suggest *SLC5A10* may be an important transporter of 1,5-AG. Conditional analysis identified multiple distinct signals in this locus. Two of the variants identified (rs61741107 and rs148178887) were also found in a whole genome sequencing analysis of a metabolome panel^20^, adding further evidence to the importance of this region in influencing 1,5-AG levels. The effect sizes of most of the *SLC5A10* variants are large. Given the distribution of 1,5-AG in this sample (in European ancestry: 3.4 to 38.2 winsorized) having just one copy of the rs61741107 or rs148178887 allele would result in a lowering of 1,5-AG by 10 µg/mL on average. Although no individuals in this dataset are homozygous for rs61741107 (1000 genomes European ancestry MAF = 0.002) or rs148178887 (1000 genomes European ancestry MAF = 0.005), the allele frequencies indicate that such individuals do exist in the population, and would have lowering of 1,5-AG levels of over 20 µg/mL on average. In addition, these effect sizes and allele frequencies were similar across ancestries. The smaller p value for the T1 gene-based test as compared to the SKAT test indicates that these variants impact 1,5-AG levels in the same direction. The similar p-value for SKAT-O when restricting variants to MAF <0.05 indicates that the relevant variants are low-frequency and rare.

Many of the variants in this region are predicted to be damaging or deleterious by multiple programs. *SLC5A10* partially overlaps with *FAM83G*, which is expressed in the skin and esophagus (eQTLs; https://www.gtexportal.org/home/)^19^. It is not likely that variants in this region represent diabetes-related factors; neither gene is known to impact diabetes risk, fasting glucose or HbA1c. In addition, variants near *SLC5A10* were not associated with diabetes status, and comparing individuals with and without variants in *SLC5A10*, multiple other measures of hyperglycemia were similar, while mean 1,5-AG differed substantially (Supplementary Fig. S2). Given this evidence, it is likely that rs61741107 and rs148178887 represent putative causal variants for 1,5-AG in this region.

In addition, significant, common variants on chromosomes 1, 2, 3 and 22, and a low frequency variant on chromosome 7 were associated with 1,5-AG concentrations. These loci were all identified by our previous GWAS in the region near *LCT/UBXN4/R3HDM1/RAB3GAP1*. While several of the variants in this region are nonsynonymous, they were mainly not predicted to be damaging or deleterious, and the effect sizes are relatively small, indicating a potentially more modest impact on 1,5-AG levels. Other regions which were significantly associated among Europeans but not Africans including *MGAM*, *SLC5A1* and *SI*. Further studies are needed to confirm the role of rare variants in these regions in 1,5-AG.

There is currently debate about the utility of 1,5-AG as a useful biomarker of hyperglycemia in adults with diabetes. Prior to widespread use of any clinical test, it is important to identify limitations overall or for specific subpopulations. Warren *et al*. have shown that there is a proportion of individuals for whom 1,5-AG produces “false positive results”, i.e., where 1,5-AG concentrations are low while fasting glucose and 2-hour glucose levels are not elevated^21^. Our work shows evidence of a strong genetic impact on 1,5-AG unrelated to diabetes, which may explain some of these findings. The likely nonglycemic genetic impact on 1,5-AG identified in this work is similar to previous findings in HbA1c, for which variants have been identified which impact HbA1c levels, but are not important mechanisms of glucose control^9,22,23^. Our results may have implications for the overall utility of the biomarker independent of genetic characterization.

Exome sequencing has highlighted the role of *SLC5A10* influencing 1,5-AG levels. This study has provided insight into the biology of this biomarker. Although these rare variants impact a smaller number of individuals than common variants, the large effect sizes would likely alter 1,5-AG levels in a sufficient manner to substantially impact its usefulness as a biomarker of hyperglycemia for carriers.

## Supplementary information

Supplementary tables and figures

## Data Availability

The datasets generated during and/or analyzed during the current study are available in the dbGAP repository, https://www.ncbi.nlm.nih.gov/projects/gap/cgi-bin/study.cgi?study_id=phs000668.v2.p1.
