# Supplementary material for: Rare variants in SLC5A10 are associated with serum 1,5-anhydroglucitol (1,5-AG) in the Atherosclerosis Risk in Communities (ARIC) Study
Source: Sci Rep. 2019 Apr 11;9:5941. doi: 10.1038/s41598-019-42202-0 (PMC6459884; doi:10.1038/s41598-019-42202-0)
Supplement: Supplementary file 1 — Supplementary tables and figures [file 41598_2019_42202_MOESM1_ESM.docx]

**Supplementary tables and figures**

**TITLE:** Rare variants in *SLC5A10* are associated with serum 1,5-anhydroglucitol (1,5-AG) in the Atherosclerosis Risk in Communities (ARIC) Study

**AUTHORS:**

Stephanie J. Loomis^1^, sloomis5@jhu.edu

Anna Köttgen^1,2^, anna.koettgen@uniklinik-freiburg.de

Man Li^1,3^, man.li@hsc.utah.edu

Adrienne Tin^1^, atin1@jhu.edu

Joesf Coresh^1,4^, coresh@jhu.edu

Eric Boerwinkle^5^, eric.boerwinkle@uth.tmc.edu

Richard Gibbs^6^, agibbs@bcm.edu

Donna Muzny^6^, donnam@bcm.edu

James Pankow^7^, panko001@umn.edu

Elizabeth Selvin^1, 4^, eselvin@jhu.edu

Priya Duggal^1^*, pduggal@jhu.edu

Elizabeth Selvin and Priya Duggal contributed equally to this work.

^1^Department of Epidemiology, The Johns Hopkins University Bloomberg School of Public Health, Baltimore MD

^2^Institute of Genetic Epidemiology, Medical Center and Faculty of Medicine - University of Freiburg, Freiburg, Germany

^3^Division of Nephrology and Department of Human Genetics, University of Utah, Salt Lake City, Utah

^4^Welch Center for Prevention, Epidemiology, & Clinical Research, The Johns Hopkins University, Baltimore MD

^5^Department of Epidemiology, The University of Texas Health Science Center at Houston School of Public Health at Houston, Houston, TX

^6^Human Genome Sequencing Center, Baylor College of Medicine, Houston, TX

^7^Division of Epidemiology and Community Health, University of Minnesota, Minneapolis, MN

**CORRESPONDING AUTHOR:**

Priya Duggal

Email: pduggal@jhu.edu

Phone: 410-955-1213

**Supplemental Table S1**. Study population characteristics.^a^

|  | **European ancestry (N=6,589)** | **African ancestry (N=2,309)** | **Overall (N=8,898)** |
| --- | --- | --- | --- |
| Female | 55% | 63% | 57% |
| Age | 57 (5.6) | 56 (5.7) | 56.7 (5.7) |
| Fructosamine (µmol/L) | 227 (23) | 238 (32) | 230 (26) |
| Glycated albumin (%) | 12.6 (1.6) | 13.6 (2.4) | 12.9 (1.9) |
| 1,5-AG (µg/mL) | 18.9 (5.8) | 17.5 (5.8) | 18.5 (5.8) |
| HbA1c (%) | 5.4 (0.52) | 5.8 (0.86) | 5.5 (0.64) |
| Fasting glucose (mg/dL) | 104 (17) | 109 (26) | 105 (20) |
| ARIC study center |  |  |  |
| Jackson, Mississippi | 0 | 90% | 23% |
| Forsyth Co, North Carolina | 25% | 10% | 21% |
| Washington Co, Maryland | 30% | 0 | 23% |
| Minneapolis suburbs, Minneapolis | 45% | 0 | 33% |

^a^Continuous variables shown as mean (SD) and categorical variables shown as (%).

**Supplemental Table S2**. Significant^a^ 1,5-AG (µg/mL) T1 gene-based results in European ancestry sample, validated in African ancestry sample.

|  |  | **European ancestry (N=6,589)** | | | | **African ancestry (N=2,309)** | | | | **Meta-analysis** | |
| --- | --- | --- | --- | --- | --- | --- | --- | --- | --- | --- | --- |
| Chr | Gene | P-value | Beta | cMAF | N SNPs | P-value | Beta | cMAF | N SNPs | P-value | Beta (SE) |
| 17 | SLC5A10 | 2.5E-114 | -8.65 (0.38) | 0.017 | 57 | 7.5E-07 | -2.99 (0.60) | 0.019 | 26 | 1.80E-88 | -5.74 (0.29) |
| 17 | FAM83G | 1.3E-14 | -2.67 (0.35) | 0.016 | 53 | 0.25 | 0.47 (0.40) | 0.04 | 35 | 7.19E-05 | -0.81 (0.20) |
| 3 | SI | 6.4E-07 | 1.25 (0.25) | 0.039 | 130 | 0.14 | 0.46 (0.31) | 0.07 | 63 | 2.73E-05 | 0.68 (0.16) |

a. Bonferroni corrected significance threshold = 2.0x10^-6^ (0.05/(12,504 genes * 2 gene-based tests)).

**Supplemental Table S3**. Significant^a^ 1,5-AG (µg/mL) SKAT gene-based results in European ancestry sample, validated in African ancestry sample.

|  |  | **European ancestry (N=6,589)** | | | **African ancestry (N=2,309)** | | | **Meta-analysis** |
| --- | --- | --- | --- | --- | --- | --- | --- | --- |
| Chr | Gene | P-value | cMAF | N SNPs | P-value | cMAF | N SNPs | P-value |
| 17 | SLC5A10 | 2.8E-55 | 0.038 | 58 | 6.5E-03 | 0.29 | 28 | 7.03E-83 |
| 17 | FAM83G | 2.8E-18 | 0.060 | 56 | 0.60 | 0.34 | 36 | 4.50E-17 |
| 7 | MGAM | 7.5E-09 | 0.087 | 148 | 0.03 | 0.95 | 98 | 6.57E-06 |
| 22 | SLC5A1 | 6.0E-07 | 0.227 | 48 | 0.11 | 0.07 | 15 | 1.27E-07 |

^a^Bonferroni corrected significance threshold= 2.0x10^-6^ (0.05/(12,504 genes * 2 gene-based tests)).

**Supplemental Table S4.** Significant^a^ 1,5-AG (µg/mL) gene-based SKAT-O results in European ancestry sample restricting variants to MAF<0.05.

| Chr | Gene | P-value | cMAF^b^ | N SNPs |
| --- | --- | --- | --- | --- |
| 17 | SLC5A10 | 5.13E-64 | 0.04 | 58 |
| 17 | FAM83G | 6.24E-17 | 0.06 | 56 |
| 7 | MGAM | 8.20E-07 | 0.09 | 148 |

a. Bonferroni corrected significance threshold = 4.4x10-6 (0.05/11,332 genes).

b. cMAF=cumulative minor allele frequency

**Supplemental Table S5.** Association between chromosome 17 SNPs and diabetes status^a^

|  | **European ancestry (N=6,998)** | | | | **African ancestry (N=2,704)** | | | |  |
| --- | --- | --- | --- | --- | --- | --- | --- | --- | --- |
|  | **Diagnosed diabetes**  **(N=409 cases)** | | **Diagnosed and undiagnosed diabetes**  **(N=760 cases)** | | **Diagnosed diabetes**  **(N=395 cases)** | | **Diagnosed and undiagnosed diabetes**  **(N=644 cases)** | | |
|  | OR (95% CI) | P-value | OR (95% CI) | P-value | OR (95% CI) | P-value | OR (95% CI) | P-value | |
| rs61741107^b^ | 0.85 (0.34, 2.12) | 0.73 | 0.95 (0.49, 1.85) | 0.88 | -- | -- | -- | -- | |
| rs148178887 | 0.34 (0.05, 2.50) | 0.29 | 1.41 (0.63, 3.19) | 0.41 | 1.61 (0.18, 14.55) | 0.67 | 0.85 (0.09, 7.71) | 0.89 | |
| rs117355297 | 1.01 (0.69, 1.47) | 0.98 | 1.03 (0.77, 1.34) | 0.85 | 0.96 (0.33, 2.81) | 0.95 | 1.06 (0.45, 2.51) | 0.90 | |

a. Diagnosed diabetes is defined as self-reported physician diagnosis or use of diabetes medications. Undiagnosed diabetes is defined as fasting glucose ≥126 mg/dL if fasting for ≥8 hours or fasting glucose >200 if not fasting for ≥8 hours).

b. Only two African ancestry individuals had rs61741107 alleles and thus was too colinear with diabetes to produce a regression estimate.

**Supplemental Table S6**. Chromosome 2 significant results in European ancestry individuals, unconditioned and conditioned on top nonsynonymous variants.

|  |  |  |  |  | **Unconditioned** | | **Conditioned on rs961360** | | **Conditioned on rs961360 and rs2305165** | |
| --- | --- | --- | --- | --- | --- | --- | --- | --- | --- | --- |
| SNP | Gene | Position | A1/A2^b^ | Effect AF | Beta (SE) | P-value^1^ | Beta (SE) | P-value^1^ | Beta (SE) | P-value^a^ |
| rs961360 | *R3HDM1* | 136393658 | A/G | 0.15 | -0.80 (0.14) | **7.82E-09** | -- | -- | -- | -- |
| rs2305165 | *R3HDM1* | 136409574 | A/C | 0.08 | -0.85 (0.18) | 1.86E-06 | -0.98 (0.18) | **5.89E-08** | -- | -- |
| rs2304371 | *LCT* | 136561557 | G/A | 0.83 | 0.89 (0.13) | **6.74E-12** | 0.79 (0.20) | 1.2E-04 | 0.58 (0.21) | 0.006 |
| rs3739022 | *LCT* | 136562472 | G/A | 0.10 | -1.07 (0.17) | **1.23E-10** | -1.02 (0.17) | **8.99E-10** | -0.78 (0.22) | 4.4E-04 |
| rs1050115 | *UBXN4* | 136511817 | A/G | 0.15 | -0.80 (0.14) | **5.69E-09** | -0.53 (0.27) | 0.05 | -0.14 (0.28) | 0.61 |
| rs10445686 | *RAB3GAP1* | 135893372 | A/G | 0.13 | -0.79 (0.14) | **3.59E-08** | -0.35 (0.24) | 0.14 | -0.23 (0.24) | 0.33 |

a. Bold indicates exome-wide significance (Bonferroni corrected significance threshold = 4.1x10^-7^ (0.05/121,052 SNPs)).

b. A2 is effect allele

**Supplemental Figure S1**. Sample exclusions

**Supplemental Figure S2**. Distribution of biomarkers by chromosome 17 variants.^a,b^


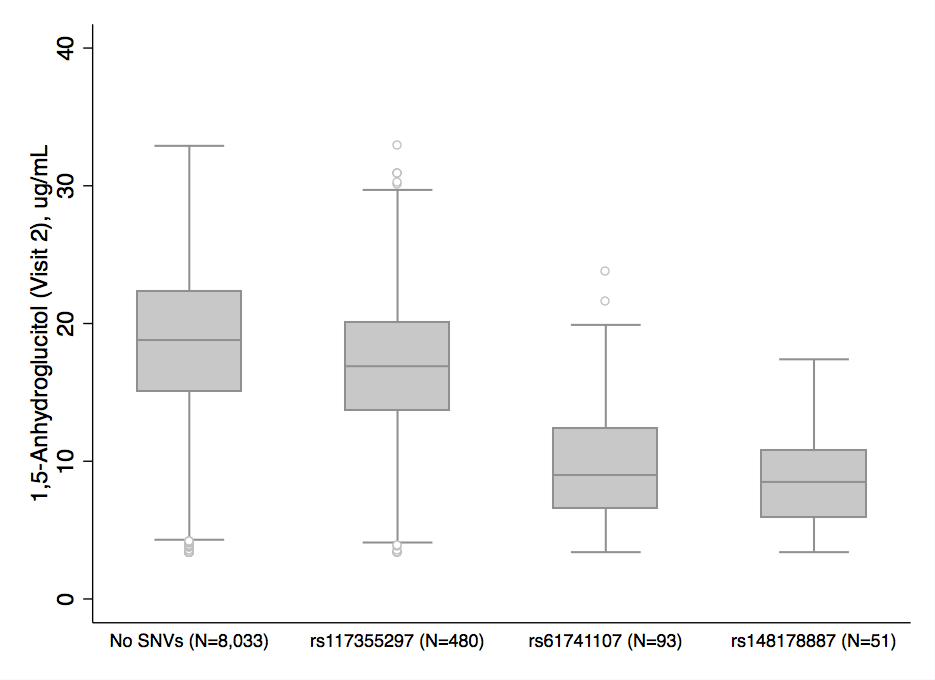

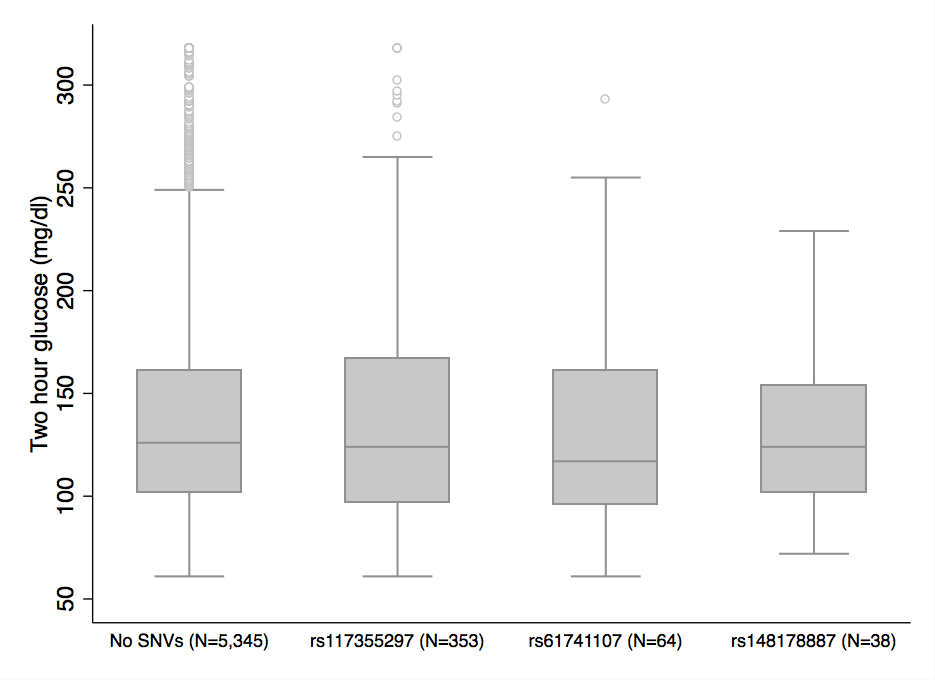


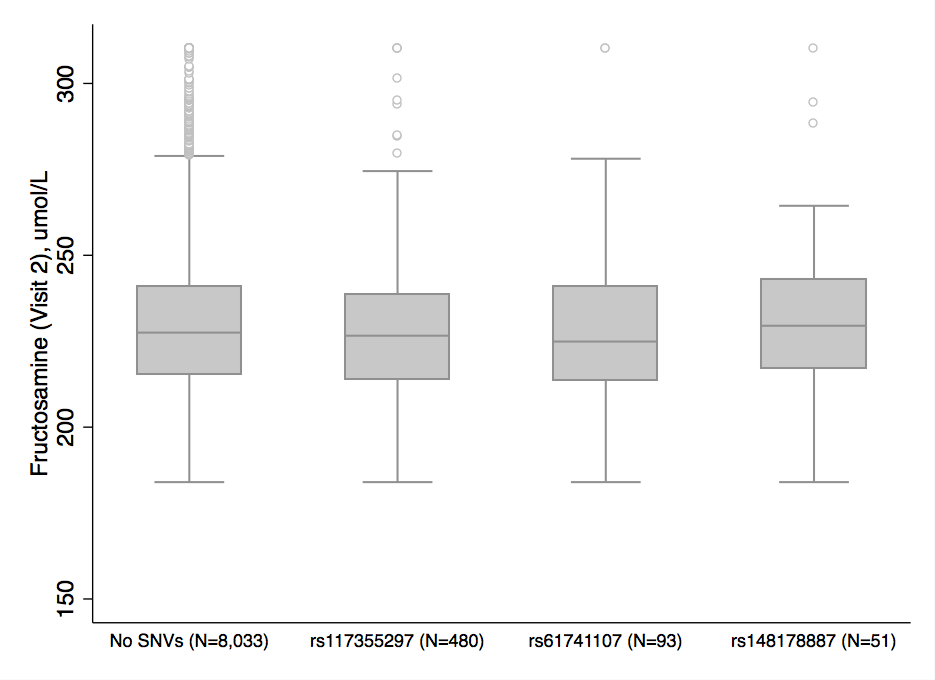

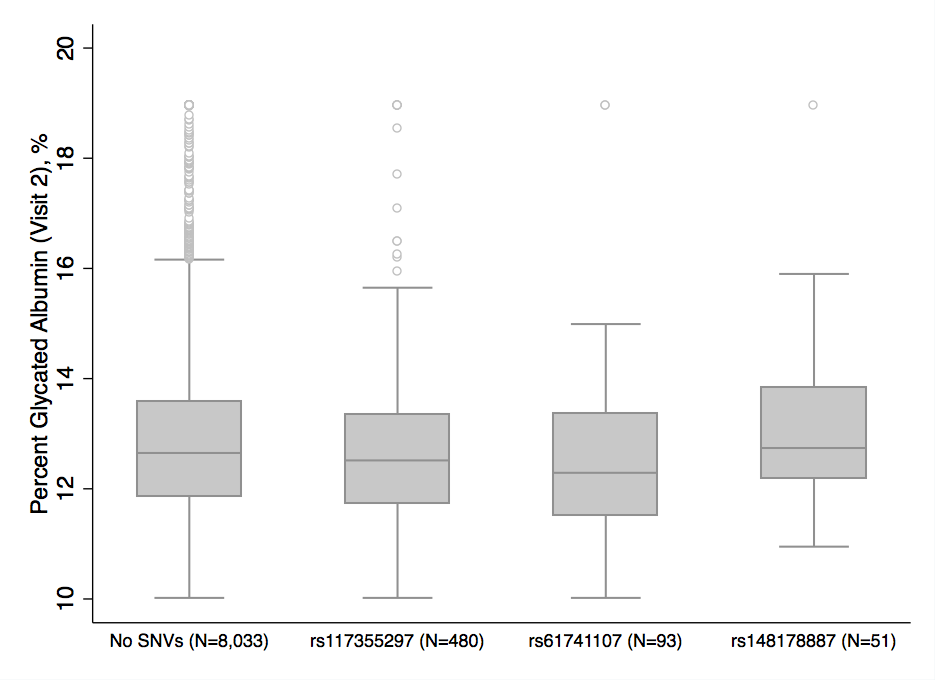

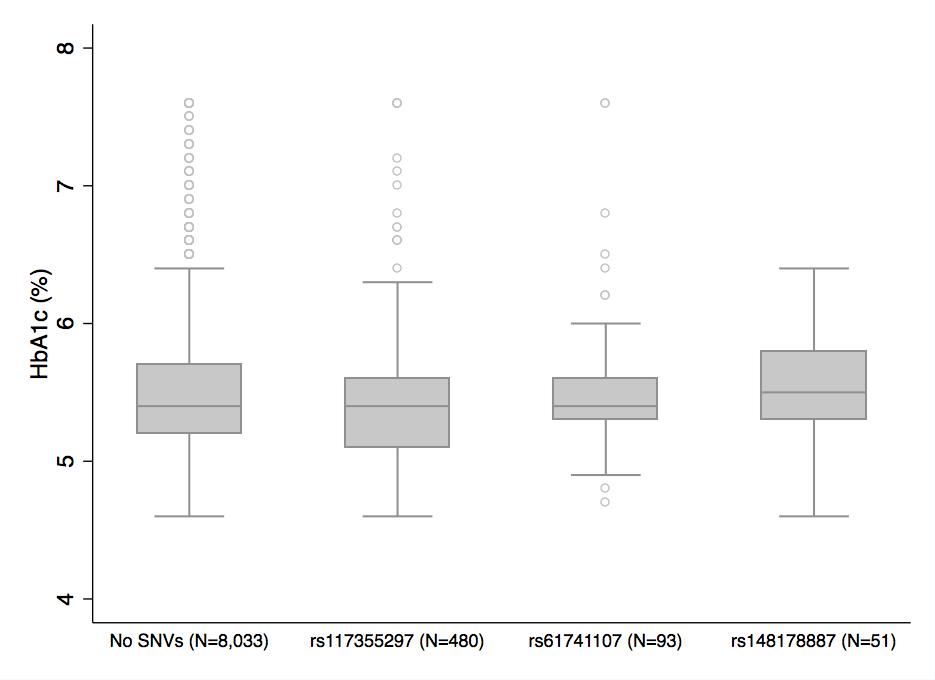

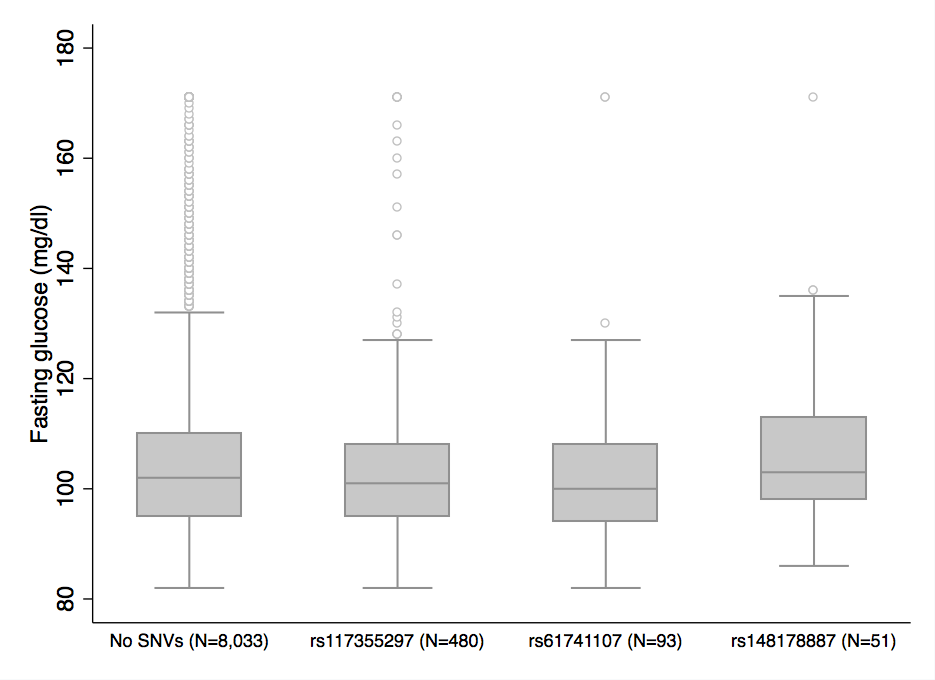


a. Biomarkers are winsorized at 1% and 99%.

b. Two hour glucose was measured at visit 4, other biomarkers were measured at visit 2.

**Supplemental Figure S3.** Distribution of 1,5-AG by chromosome 17 variant genotypes.

**
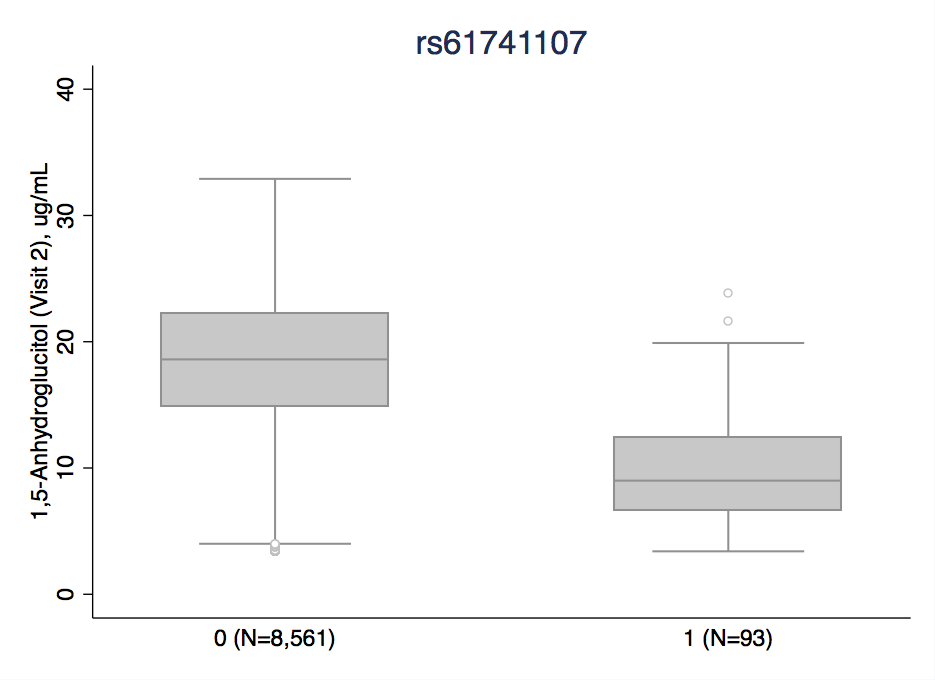

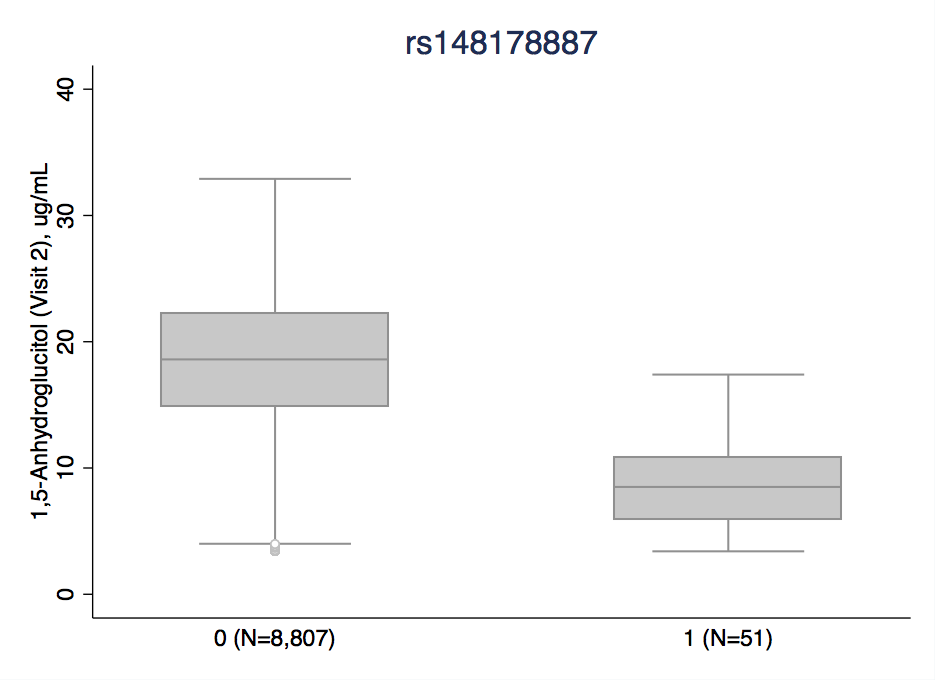

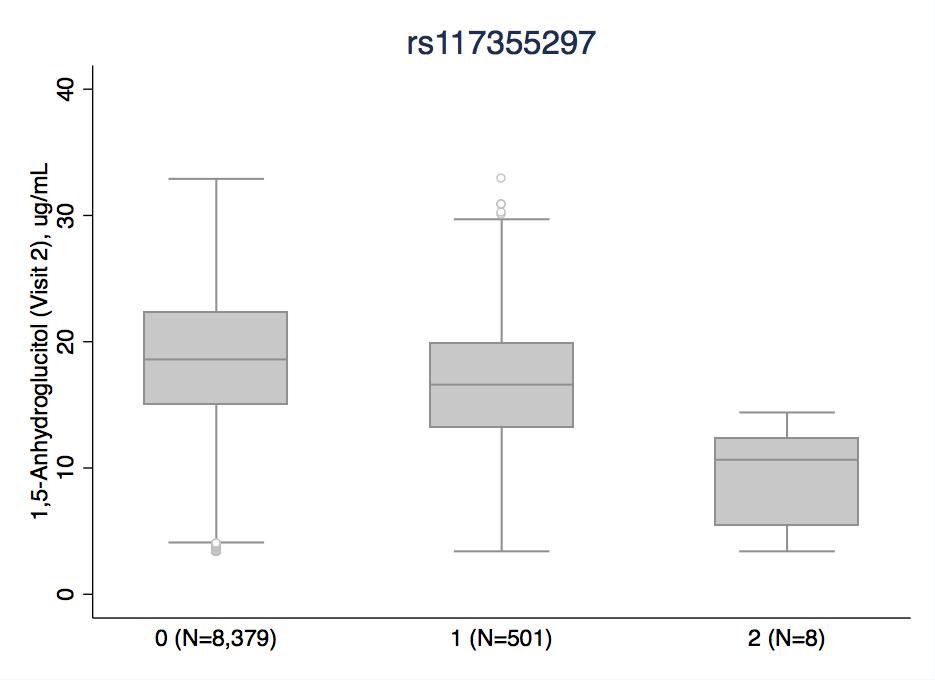
**
